# Artificial intelligence for predicting post-excision recurrence and malignant progression in oral potentially malignant disorders: a retrospective cohort study

**DOI:** 10.1097/JS9.0000000000003592

**Published:** 2025-10-07

**Authors:** John Adeoye, Yu-Xiong Su

**Affiliations:** a Division of Applied Oral Sciences and Community Dental Care, Faculty of Dentistry, University of Hong Kong, Hong Kong SAR, China; b Division of Oral and Maxillofacial Surgery, Faculty of Dentistry, University of Hong Kong, Hong Kong SAR, China

**Keywords:** artificial intelligence, lesion recurrence, malignant progression, oral cancer, oral precancerous conditions

## Abstract

**Background:**

Oral cancer may develop from precursor lesions and conditions termed oral potentially malignant disorders (OPMD), although not all patients progress to cancer in their lifetime. Managing patients with OPMD is challenging due to lesion recurrence and uncertain malignant progression risk following surgical excision. This study developed a multitask AI-based model to predict the risk of treatment failure, malignant progression, and recurrence among patients with OPMD treated by surgery.

**Methods:**

This study utilized multidimensional data from 366 retrospective patients with OPMD treated in 2 tertiary centers to construct an AI model to predict 3 treatment outcomes among patients with OPMD. Multifaceted prognostic variables were collected for the cohort and used to train four AI supervised learning models, followed by optimal model selection. Area under the receiver operating characteristic curve (AUC) and Brier scores (BSs) were used to assess model performance. External testing of the model was also performed, and metrics were compared to the WHO and binary dysplasia grading systems (current standards). We further assessed the net benefit and explainability of the final multitask model.

**Results:**

The outperforming model (TabPFN) had good AUC values of 0.829 (0.729–0.929), 0.912 (0.836–0.988), and 0.791 (0.683–0.899) for predicting treatment failure, malignant progression, and lesion recurrence at external testing. The BSs of the model for all three treatment outcome predictive tasks were also optimal (0.085–0.147). Furthermore, the AI model had a superior net benefit than the WHO and binary epithelial dysplasia grading systems in assessing the need for close monitoring among patients with OPMD treated by surgery. The explainability of the model was also successfully implemented.

**Conclusions:**

The multitask AI-based model developed with multidimensional data has good discriminatory performance, calibration, and net benefit, showing potential for comprehensive risk assessment and clinical decision support in the surgical management of patients with OPMD to promote early detection of oral cancer.

**HIGHLIGHTS**

- Developed a multitask AI-based model with multidimensional data for the prediction of treatment failure, malignant progression, and lesion recurrence among patients with OPMD.
- The AI model had good discriminative performance and calibration for predicting the three treatment outcomes.
- Model had superior benefit than the WHO and binary epithelial dysplasia grading systems (current methods) in selecting surgical patients with OPMD that require close follow-up.
- The explainability of the methods was successfully implemented to ensure transparency.

## Introduction

Oral cancer is the most common head and neck malignancy globally, the majority of which are squamous cell carcinomas^[1,2]^. The 5-year survival rate of oral cancer has been approximately 50% for many years[3], which may be considered poor given that the mouth is accessible for visual and tactile clinical examination. Early detection of malignant disease is crucial to improving tumor prognosis by more than 30%^[4,5]^. Often, oral cancer is preceded by precursor lesions termed oral potentially malignant disorders (OPMD)[6]. OPMD are diverse mucosal conditions with a significantly increased risk of malignant progression, and as such, their detection and management are crucial to oral cancer prevention and early detection[6].

Among OPMD lesions, white or mixed white and red mucosal lesions such as oral leukoplakia, lichen planus, and lichenoid lesions are more common than other subtypes^[6,7]^. Surgical excision is the mainstay treatment of many OPMDs, especially leukoplakia, and lichenoid disease with dysplasia; however, intervention is not recommended for all patients and depends on cancer risk assessment[8]. Managing OPMD may also be challenging due to disease recurrence[9]. Recurrent lesions have been found to have a significantly increased risk of cancer progression than nonrecurrent OPMD^[9,10]^.

Presently, there is no standard method for assessing the risk of surgical recurrence in OPMD. Moreover, while epithelial dysplasia (ED) grading is the current standard for cancer risk assessment of OPMD[11], the method is unidimensional, has variable accuracy, lacks reproducibility, and does not apply to OPMD patients without ED, which is impractical given that some of these patients progress to malignancy^[12,13]^.

Artificial Intelligence (AI) involves using contemporary techniques such as machine learning to recognize patterns that are key to downstream predictions from data, making them appropriate for constructing accurate and actionable models for clinical tasks to provide decision support for patient management^[12,14–16]^. However, models trained on state-of-the-art (SOTA) deep learning architectures and multivariate data (obtained from surgical cohorts with long-term follow-up) for the comprehensive assessment of patients with OPMD are limited. Therefore, in this study, we constructed a multitask AI-based risk stratification model (using a tabular prior-data fitted network) for synchronous prediction of treatment failure, malignant progression, and lesion recurrence among patients with OPMD treated by surgical excision. This study adhered to the Strengthening the reporting of cohort, cross-sectional, and case control studies in surgery (STROCSS) guideline in its reporting[17].

## Methods

### Patient data and outcomes

This study involved 312 retrospective patients with OPMD treated via surgical excision with curative intent at Queen Mary Hospital (QMH) and Prince Philip Dental Hospital (PPDH), Hong Kong, from January 2003 to December 2020. Specifically, patients with OPMD included in this study had oral leukoplakia and oral lichenoid diseases. These clinical OPMD subtypes were investigated since their malignant progression proportions vary significantly from 0.3 to 41%^[18–20]^, which is uninformative during surgical treatment and disease surveillance. The inclusion criteria were based on the clinical and histological case definition of OPMD according to the WHO 2020 criteria[6]. Patients with other OPMD, such as erythroplakia and proliferative verrucous leukoplakia, or those with previous oral cancer, were excluded.

Three outcomes were considered in this study, which included (surgical) treatment failure, malignant progression, and lesion recurrence. Lesion recurrence was defined as the reappearance of OPMD at the same anatomic site following surgical excision of primary lesions with curative intent. Malignant progression also meant the development of biopsy-proven squamous cell carcinoma from the lesion site at or after lesion recurrence (including after multiple recurrences), which was determined as of May 1, 2025. Surgical treatment was deemed to have failed if patients developed OPMD relapse or cancer following surgical excision.

### Multidimensional data and preprocessing

Multifaceted prognostic features independently associated with lesion recurrence and cancer progression, according to previous reports, were collected for all patients in the cohort^[10,21,22]^. These included demographics (age and sex), risk habits (tobacco use and alcohol drinking), medical history (previous cancer), clinical features (anatomic size, homogeneity, induration, and number of lesions), treatment information (surgical modality and margin involvement), and histopathologic characteristics (lichenoid features and dysplasia status). Positive (incomplete) margin status was defined by the presence of histologic features of the lesion, such as dysplasia or hyperplasia, within 1 mm of the surgical margins, and other lesions were classified as negative margins (complete excision). Binary outcomes indicating treatment failure, recurrence status, and malignant progression at any time were collected. No missing instances were present in any of the variables. Since patients may have OPMD at multiple anatomic sites, one-hot encoding was performed for the sites affected. Additionally, we performed label encoding of the other categorical variables.

### Model construction

We selected two SOTA deep learning architectures for structured data [Tabular Prior-fitted data network (TabPFN)[23] and TabNet[24]] and benchmarked them against two traditional tree-based machine learning classifiers – XGBoost[25] and RF[26] for multitask prediction of treatment outcomes among patients with OPMD treated by surgery. Prior-fitted data networks (PFNs) are frameworks that perform in-context learning and leverage Bayesian inference to sample from a prior distribution over supervised learning tasks with minimal tuning[23]. Using this approach, a transformer-based pre-trained framework that approximates a posterior predictive distribution according to training data and trained offline using meta-learning could be adapted to new training sets to predict class probabilities[27]. Recently, PFNs have been extended to tabular data by adjusting transformer modules to one- and two-dimensional data in train–test split tables, with this architecture trained to solve over 100 million tasks[28]. As such, we adapted this foundational model for multitask prediction of treatment failure, malignant progression, and OPMD recurrence in this study, comparing it to existing deep and traditional machine learning models for structured data.

For model training, nested 10-fold cross-validation (CV) was implemented for hyperparameter tuning (TabNet, XGBoost, and RF), model stability, and internal performance analysis. Hyperparameters selected for each model are listed in Supplementary Digital Content, Table S1, available at: http://links.lww.com/JS9/F281. Class imbalance was also addressed by specifying class weights for the comparator models. Performance metrics assessed at training included area under the receiver operating characteristic curve (AUC) and Brier score (BS), which evaluated the discriminative ability and calibration of the models.

### External testing

To assess generalizability, external testing was performed using data from 54 patients with OPMD treated by surgery at PPDH, from January 2021 to December 2023. Patients were followed till 1 May 2025, and similar prognostic factors as the training cohort were collected. Discrimination and calibration of the models were evaluated using AUC and BS and compared to those of the WHO and binary dysplasia grading, as the current methods for assessing OPMD cancer risk. The final model was selected based on the external testing discriminative and calibration performances for all three treatment outcomes. Additionally, stratified performance analysis was used to evaluate algorithmic bias based on sex, lichenoid features, and dysplasia status for treatment failure, malignant progression, and lesion recurrence. We also conducted an analysis to determine the characteristics of patients likely to be assigned an inaccurate predicted probability by the final model. Global and local explanations of the model were implemented using Shapley additive explanations (SHAP) on the external testing dataset. Decision curve analysis was also conducted to estimate the net benefit of the model at threshold probabilities below 50%. The decision curve of the optimal AI model was also compared to those of the WHO and binary ED grading systems and reference decision curves.

### Statistics and computation

Comparison between patients with different treatment outcomes based on prognostic variables was conducted using the Kruskal–Wallis test for continuous data and the Chi-square test or Fisher’s exact test for categorical variables. Probability values below 5% were considered statistically significant. Statistical analysis and plots were conducted using SPSS v28 and GraphPad Prism. Model training was done using Python v3.10.

### Ethics

Approval to conduct this study was granted by the Institutional Review Board of the University of Hong Kong/Hospital Authority Hong Kong West Cluster (Reference no: UW 22-789). Informed consent was waived for this study due to the use of secondary data. However, all potential patient identifiers were masked before data analysis.

## Results

### Patient cohort

**Figure 1.**
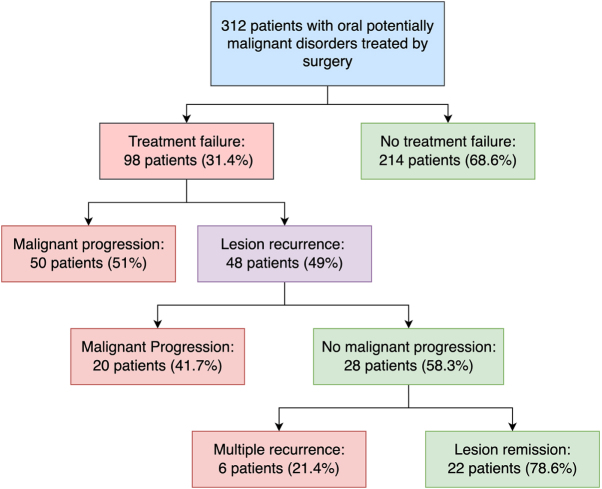
Flowchart showing the treatment outcomes of patients in the OPMD surgical cohort used for AI model training.

The distribution of multidimensional prognostic factors collected for patients with OPMD is in Table 1. The median age of the cohort was 60 years, with slightly more females than males (53.2% vs. 46.8%). Notably, 21.5% (67) of patients had OPMD with dysplasia, and 9% were classified as high-grade according to the binary dysplasia grading system. Patients were followed for a median time of 96.5 and 107 months for OPMD treatment failure and malignant progression, respectively. Treatment outcomes are in Figure 1. In detail, 98 patients (31.4%) experience treatment failure, with 70 patients having lesions that (22.4%) progressed to oral cancer. Furthermore, 15.4% of patients (48) had lesion recurrence alone or before malignant progression. Patients were followed for a median time of 96 months for OPC recurrence and 109 months for malignant progression.

**Table 1:** Demographic characteristics of the surgical cohort used for AI model construction to predict treatment failure, malignant progression, and lesion recurrence among patients with OPMD

Prognostic factors	Labels	Training cohort *n* (%)	External validation cohort *n* (%)
Age	Median (IQR)	60 (51–68.8)	65 (57–69)
Sex	Female	166 (53.2)	33 (61.1)
	Male	146 (46.8)	21 (38.9)
Risk habit category	Non-smoker and non-drinker	212 (67.9)	37 (68.5)
	Smoker and/or drinker	100 (32.1)	17 (31.5)
Previous cancer	Solid tumors – Head and neck	11 (3.5)	6 (11.1)
	Solid tumors – Others	30 (9.6)	6 (11.1)
	Hematological malignancy	13 (4.2)	1 (1.9)
	No malignancy	258 (82.7)	41 (75.9)
Sites affected	Tongue/floor of the mouth	178 (57.1)	28 (51.9)
	Buccal/labial mucosa	118 (37.8)	19 (35.2)
	Retromolar	6 (1.9)	5 (9.3)
	Gingiva	31 (9.9)	5 (9.3)
	Palate	12 (3.8)	3 (5.6)
Number of lesions	1	241 (77.2)	43 (79.6)
	2	47 (15.1)	8 (14.8)
	≥3	24 (7.7)	3 (5.6)
Homogeneity	Homogeneous	239 (76.6)	34 (63)
	Non-homogeneous	73 (23.4)	20 (37)
Induration	Yes	31 (9.9)	5 (9.3)
Size	Median (IQR)	0.8 (0.5–1.3)	0.8 (0.5–1)
Surgical modality	Laser excision ± vaporization	12 (3.8)	5 (9.3)
	Conventional scalpel	300 (96.2)	50 (90.7)
Excision status	Negative margin	279 (89.4)	48 (88.9)
	Positive margin	33 (10.6)	6 (11.1)
Lichenoid features on histology	No	229 (73.4)	40 (74.1)
	Yes	83 (26.6)	14 (25.9)
WHO dysplasia grading	None	245 (78.5)	38 (70.4)
	Mild	29 (9.3)	9 (16.7)
	Moderate	23 (7.4)	1 (1.9)
	Severe	15 (4.8)	6 (11.1)
Binary Dysplasia	No/low grade	284 (91)	47 (87)
	High grade	28 (9)	7 (13)
Treatment failure (*n*%)		98 (31.4)	14 (25.9)
Lesion recurrence (*n*%)		48 (15.4)	8 (14.8)
Malignant progression (*n*%)		70 (22.4)	7 (13.0)
Follow-up times for outcome assessment	Time to treatment failure a	96.5 (55–162.5)	34 (25.5–47)
	Malignant progression b	107 (68.3–172.5)	37.5 (29–47)

^a^ Time to (first) lesion recurrence or direct cancer progression following primary surgical excision of OPMD.

^b^ Time to cancer progression for patients who developed cancer at the time of treatment failure or subsequently after recurrence.

Patients were stratified based on treatment failure outcomes (Table 2), revealing significant differences in certain prognostic factors in the cohort. Compared to OPMD patients with recurrence and no malignant progression or patients with no treatment failure, those who developed cancer at treatment failure or after lesion recurrence had mostly nonhomogeneous primary lesions that involved the tongue, had positive margins on excision, and high-grade dysplasia on histology (*P* < 0.001–0.022, Table 2). Patients without treatment failure had smaller primary lesions that involved the buccal mucosa with no or low-grade dysplasia (*P* < 0.001–0.039) compared to other groups.

**Table 2:** Comparison of multidimensional prognostic variables among patients with OPMD stratified by treatment outcomes

Prognostic factors	Labels	Treatment outcomes				*P*-value
		Treatment failure			No treatment failure (*n* = 214)	
		Cancer at treatment failure (*n* = 50)	OPMD recurrence at treatment failure with subsequent cancer progression (*n* = 20)	OPMD recurrence at treatment failure with no cancer progression (*n* = 28)		
Age	Median (IQR)	63.5 (54–71)	59.5 (46–68.8)	63.5 (50.8–73.8)	59 (51–68)	0.135^a^
Sex	Female	29 (58)	14 (70)	13 (46.4)	110 (51.4)	0.318^b^
	Male	21 (42)	6 (30)	15 (53.6)	104 (48.6)	
Risk habit category	Non-smoker and non-drinker	36 (72)	16 (80)	17 (60.7)	143 (66.8)	0.474 b
	Smoker and/or drinker	14 (28)	4 (20)	11 (39.3)	71 (33.2)	
Previous cancer	Solid tumors – Head and neck	0	0	1 (3.6)	10 (4.7)	0.335 b
	Solid tumors – Others	5 (10)	2 (10)	2 (7.1)	21 (9.8)	
	Hematological malignancy	3 (6)	2 (10)	3 (10.7)	5 (2.3)	
	No malignancy	42 (84)	16 (80)	22 (78.6)	178 (83.2)	
Sites affected	Tongue/floor of the mouth	35 (70)	16 (80)	9 (32.1)	108 (50.5)	**0.005** b
	Buccal/labial mucosa	15 (30)	4 (20)	7 (25)	92 (43)	**0.039** b
	Retromolar	3 (6)	0	0	3 (1.4)	0.180^c^
	Gingiva	1 (7)	1 (5)	3 (10.7)	20 (9.3)	0.682 c
	Palate	2 (4)	0	1 (3.6)	9 (4.2)	1.000 c
Number of lesions	1	42 (84)	16 (80)	20 (71.4)	163 (76.2)	0.409 b
	2	3 (6)	4 (20)	5 (17.9)	35 (16.4)	
	≥3	5 (10)	0	3 (10.7)	16 (7.5)	
Homogeneity	Homogeneous	31 (62)	13 (65)	22 (78.6)	173 (80.9)	**0.022** b
	Non-homogeneous	19 (38)	7 (35)	6 (21.4)	41 (19.2)	
Induration	Yes	6 (12)	3 (15)	2 (7.1)	20 (9.3)	0.699 c
Size	Median (IQR)	1 (0.7–2)	1.1 (0.7–2.4)	1 (0.5–1.2)	0.7 (0.4–1)	**<0.001** a
Surgical modality	Laser excision ± vaporization	2 (4)	4 (20)	1 (3.6)	5 (2.3)	**0.009** c
	Conventional scalpel	48 (96)	16 (80)	27 (96.4)	209 (97.7)	
Excision status	Negative margin	26 (52)	16 (80)	27 (96.4)	210 (98.1)	**<0.001** c
	Positive margin	24 (48)	4 (20)	1 (3.6)	4 (1.9)	
Lichenoid features on histology	No	33 (66)	13 (65)	23 (82.1)	160 (74.8)	0.331 b
	Yes	17 (34)	7 (35)	5 (17.9)	54 (25.2)	
WHO dysplasia grade of primary lesions	None	25 (50)	6 (30)	19 (67.9)	195 (91.1)	**<0.001** c
	Mild	10 (20)	3 (15)	5 (17.9)	11 (5.1)	
	Moderate	8 (16)	8 (40)	3 (10.7)	4 (1.9)	
	Severe	7 (14)	3 (15)	1 (3.6)	4 (1.9)	
Binary dysplasia of primary lesion	No/low grade	38 (76)	13 (65)	25 (89.3)	208 (97.2)	**<0.001** c
	High grade	12 (24)	7 (35)	3 (10.7)	6 (2.8)	

^a^ Kruskal–Wallis’s test.

^b^ Chi-square test.

^c^ Fisher’s exact test.

Values in bold are statistically significant.

### Model performance at internal validation

#### Treatment failure

**Figure 2.**
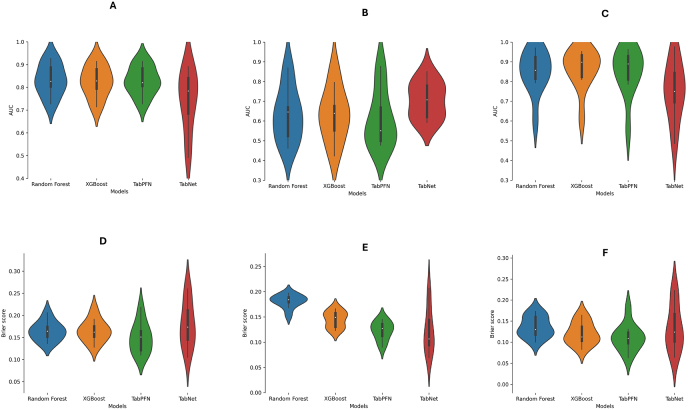
Violin plots of the cross-validation (CV) performance metrics of the AI models. (A) Model AUC plots across the 10 CV folds for predicting surgical treatment failure among OPMD patients. (B) Model AUC plots across the 10 CV folds for predicting lesion recurrence among OPMD patients. (C) Model AUC plots across the 10 CV folds for predicting malignant progression among OPMD patients. (D) Model BS plots across the 10 CV folds for predicting surgical treatment failure among OPMD patients. (E) Model BS plots across the 10 CV folds for predicting lesion recurrence among OPMD patients. (F) Model BS plots across the 10 CV folds for predicting malignant progression among OPMD patients.

Analysis revealed that XGBoost had the highest median AUC (IQR) of 0.829 (0.794–0.88) at CV compared to RF [median CV AUC: 0.826 (0.804–0.887)], TabPFN [median CV AUC: 0.821 (0.806–0.884)], and TabNet [median CV AUC: 0.784 (0.685–0.841)] (Fig. 2). However, regarding calibration and accuracy of predicted probabilities of treatment failure, TabPFN had the lowest median BS (IQR) of 0.151 (0.121–0.165), followed by XGBoost [median CV BS: 0.162 (0.152–0.175)], RF [median CV BS: 0.164 (0.152–0.174)], and TabNet [median CV BS: 0.174 (0.145–0.211)] (Fig. 2).

#### Malignant progression

Our study found XGBoost to have better discriminative ability at CV in stratifying malignant progression of OPMD treated via surgical excision [median CV AUC: 0.898 (0.823–0.934)] than other models (Fig. 2). TabPFN had the second best median AUC [0.89 (0.81–0.929)] followed by RF [0.857 (0.814–0.924)], and TabNet [0.75 (0.695–0.844)]. Regarding calibration, TabPFN had the lowest median BS [0.109 (0.098–0.123)], followed by XGBoost [0.113 (0.103–0.137)], TabNet [0.124 (0.102–0.167)], and RF [0.13 (0.12–0.16)] (Fig. 2).

#### Lesion recurrence

Median performance metrics for the models in predicting lesion recurrence were lower than for treatment failure or malignant progression at CV (Fig. 2). TabNet had better median AUC values [0.708 (0.62–0.779)] than RF [0.646 (0.524–0.669)], XGBoost [0.639 (0.553–0.676)], and TabPFN [0.551 (0.499–0.669)]. TabNet also had the lowest median BS of 0.107 (0.094–0.144). Median BSs of the TabPFN, XGBoost, and RF models were 0.127 (0.113–0.136), 0.149 (0.130–0.158), and 0.184 (0.179–0.189), respectively.

### External testing performance of AI models

**Figure 3.**
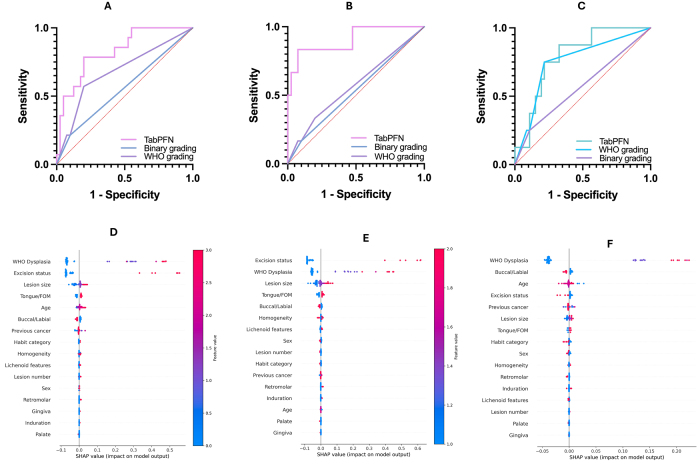
External testing performance of TabPFN multitask AI model. (A) AUC comparison of the AI model, WHO dysplasia grading, and binary grading for predicting treatment failure among OPMD patients. (B) AUC comparison of the AI model, WHO dysplasia grading, and binary grading for predicting malignant progression among OPMD patients. (C) AUC comparison of the AI model, WHO dysplasia grading, and binary grading for predicting lesion recurrence among OPMD patients. (D) SHAP summary plot to explain feature importance in predicting treatment failure. (E) SHAP summary plot to explain feature importance in predicting malignant progression. (F) SHAP summary plot to explain feature importance in predicting lesion recurrence.

TabPFN outperformed all other models in discriminative ability and calibration for predicting treatment failure, malignant transformation, and lesion recurrence among patients with OPMD treated by surgical excision at external testing (Fig. 3, Supplementary Digital Content, Figure S1, available at: http://links.lww.com/JS9/F281). The AUC (95% CI) and BS (95% CI) of the model for predicting OPMD treatment failure were 0.829 (0.729–0.929) and 0.147 (0.053–0.241), while similar metrics were obtained for stratifying malignant progression, which were 0.912 (0.836–0.988) and 0.085 (0.011–0.159). Likewise, the AUC and BS for predicting lesion recurrence were 0.791 (0.683–0.899) and 0.11 (0.027–0.193).

Beyond outperforming other AI models, the multitask TabPFN model also displayed superior risk stratification than the WHO and binary dysplasia grading systems for all three clinical tasks (Fig. 3). The WHO dysplasia grading method had AUC values of 0.681 (0.510–0.853), 0.569 (0.308–0.83), and 0.755 (0.569–0.942) while the binary dysplasia also had AUC values of 0.557 (0.375–0.74), 0.533 (0.275–0.792), and 0.571 (0.341–0.801) for predicting treatment failure, malignant progression, and lesion recurrence respectively.

### Explainability and net benefit analysis

**Figure 4.**
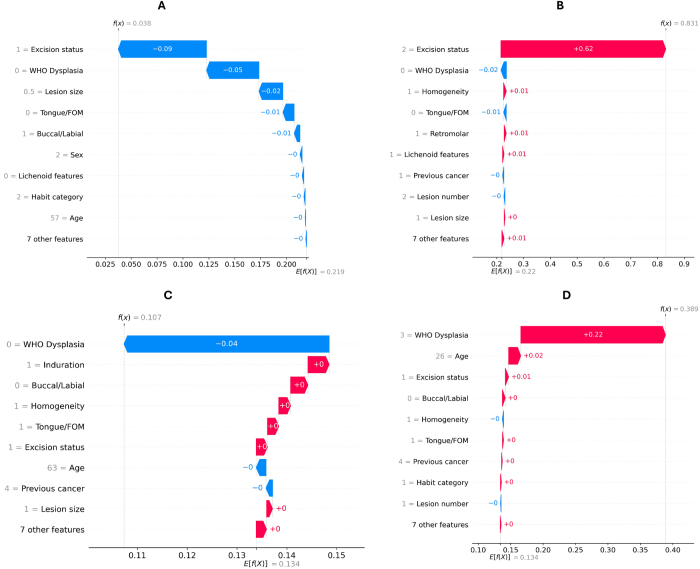
SHAP waterfall plots for local explanations of selected cases in the external testing cohort. (A) Plot showing feature importance for an OPMD patient without treatment failure. Predicted probabilities of treatment failure, malignant progression, and lesion recurrence were 11, 3.6, and 9.5%. (B) Plot showing feature importance for an OPMD patient without recurrence but with malignant progression at 31 months after diagnosis. Predicted probabilities of treatment failure, malignant progression, and lesion recurrence were 83.6, 83.1, and 10.3%. (C) Plot showing feature importance for an OPMD patient without treatment failure. Predicted probabilities of treatment failure, malignant progression, and lesion recurrence were 18.4, 11.2, and 10.7%. (D) Plot showing feature importance for an OPMD patient with recurrence at 27 months after diagnosis but no malignant progression at 45 months (last follow-up). Predicted probabilities of treatment failure and lesion recurrence were 77.1 and 38.8%.

Explainability plots of the multitask model based on global SHAP values for the external testing dataset are in Figure 3. Of note, older patients with higher dysplasia grades, positive margins, large lesions (>1 cm), tongue or floor of the mouth lesions had a high probability of treatment failure. Excision status and dysplasia grading were the top features predictive of malignant progression, while lesions with higher dysplasia grades not involving the buccal or labial mucosa had higher probabilities of lesion recurrence. Local explanation plots for specific cases with different outcomes are also presented in Figure 4.

**Figure 5.**
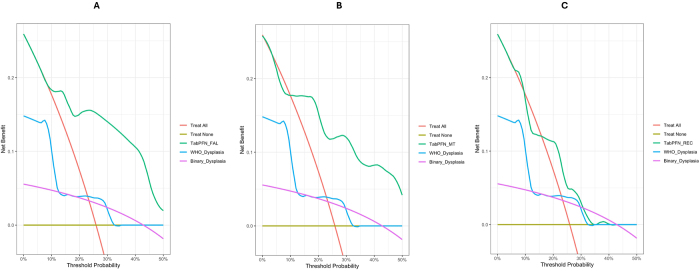
Comparing decision curves of the TabPFN model, WHO dysplasia grading, and binary dysplasia grading. (A) Decision curves showing higher net benefit for using TabPFN model over WHO and binary dysplasia grading to guide decisions on patient monitoring regarding treatment failure. (B) Decision curves showing higher net benefit for using TabPFN model over WHO and binary dysplasia grading to guide decisions on patient monitoring regarding the risk of malignant progression. (C) Decision curves showing higher net benefit for using TabPFN model over WHO and binary dysplasia grading to guide decisions on patient monitoring regarding the risk of surgical recurrence.

Decision curve analysis largely showed that the multitask TabPFN model had higher decision curves and potential clinical benefit than the WHO and binary dysplasia grading systems and reference decision curves for treatment failure, malignant progression, and lesion recurrence prediction. However, the superiority of the potential net benefit of the model (compared to existing methods) in deciding OPMD patients that need close monitoring following surgery was more marked for assessing OPMD treatment failure and cancer development than lesion recurrence (Fig. 5).

### Algorithmic bias

Stratified analysis based on sex revealed that the model performed better among male than female patients with OPMD. The AUC and BS of the model were 0.778 and 0.174 among females and 0.882 and 0.103 among males for predicting surgical treatment failure. A similar pattern in performance was also observed for malignant progression risk stratification, where the AUC and BS values for females were 0.85 and 0.124, while the values for males were 1 and 0.025. AUC and BS for lesion recurrence prediction were 0.757 and 0.113 among males and 0.852 and 0.105 among females.

When stratified by dysplasia status, the AI-based model performed better among patients without dysplasia than those with dysplasia for all three outcomes, and the difference was more marked for lesion recurrence prediction. The AUC values of the multitask model for all-time malignant progression prediction among patients with and without dysplasia were 0.872 (BS: 0.217) and 0.919 (BS: 0.03).

Based on lichenoid appearance on histopathology, which may stratify OPMD subtypes, we observed that the model performed better for patients with than without lichenoid features. For surgical treatment failure prediction, AUC values of 0.909 (BS: 0.081) and 0.799 (0.169) were observed among patients with and without lichenoid features. The model’s performance improved to an AUC of 0.97 (BS:0.059) and 0.868 (BS: 0.094) for malignant progression prediction among patients with and without lichenoid features.

### Misclassifications

Misclassification analysis was conducted based on the predicted probabilities of lesion recurrence for OPMD patients treated via surgery, since the model underperformed in this task compared to others. Patients without recurrence (within follow-up time, *n* = 9) that had a high predicted probability by the model were mostly females with solitary tongue lesions (88.9%) that had at least mild dysplasia (100%) and no lichenoid appearance (77.8%) on histology. Also, 22.2% (two) of these patients had positive margins following histopathological assessments. False-negative patients with low predicted probability of recurrence included two patients with buccal mucosa and palatal lesions without dysplasia or lichenoid features.

## Discussion

This study developed a multitask AI-based model using the TabPFN framework and multidimensional data for comprehensive assessment of the risk of treatment failure, malignant progression, and lesion recurrence among patients with OPMD treated by surgical excision. The model displayed good performance for the three clinical tasks with AUC and BS values of 0.829 (0.729–0.929) and 0.147 (0.053–0.241) for treatment failure prediction, 0.912 (0.836–0.988) and 0.085 (0.011–0.159) for malignant progression stratification, and 0.791 (0.683–0.899) and 0.11 (0.027–0.193) for predicting lesion recurrence. Furthermore, the interpretable model had better discriminative ability and net benefit compared to the WHO and binary dysplasia grading systems.

This study involved a large multicenter cohort of patients with OPMD treated by surgery and represents the first attempt to create a multitask AI-based model for treated patients to guide clinical decisions on monitoring and disease surveillance strategies that promote optimal disease prevention and early detection of oral cancer. The strength of this study is in using multifaceted prognostic variables as input features, robust treatment outcomes, SOTA deep learning architecture, strong modeling approach, external validation, and all-around performance assessment. Moreover, this study developed a unique AI model capable of predicting OPMD recurrence following surgical intervention.

Notably, our findings showed that the model performed better for stratifying the risk of malignant progression and treatment failure than for lesion recurrence. The prognostic variables used for training the multitask TabPFN model have been reported previously as independent factors associated with both lesion recurrence and malignant progression of OPMD^[9,12,21]^. Our results allude to the differences in factors that may be implicated in malignant progression and lesion recurrence, corroborating similar reports^[29,30]^. Even for our multitask model, the importance of the features varied markedly for both tasks. As such, there is a need for the discovery of new and precise predictive features, such as molecular information, immunohistochemical markers, or surgery-based factors, for optimal lesion recurrence prediction using AI frameworks. Nonetheless, the majority of patients who experienced recurrence in the external testing dataset had high predicted probabilities, which would have warranted close monitoring, indicating that false-positive cases (at last follow-up) may have influenced the discriminatory performance (AUC) of the model for predicting lesion recurrence compared to malignant progression. We will continue to follow up with these cases closely for lesion recurrence to aid future performance reporting.

Though our model uniquely predicts malignant progression for patients with OPMD treated by surgery, its performance for this task compares favorably (in terms of discrimination, calibration, and net benefit) to existing models developed that do not account for treatment types[31]. OPMD lesions are surgically excised for cancer prevention and early detection through adequate monitoring and clinical follow-up of patients[32]. As such, we maintain that the model’s key potential clinical application is to streamline the care of OPMD patients who require close monitoring following surgical excision, rather than for patients administered chemopreventive therapies or observation. With this approach, patients who need additional treatments may be promptly identified, and early malignant diseases may be detected at follow-up. Notably, we identified that the presence of lesions with positive margins following surgical excision with curative intent was predictive of malignant progression than lesion recurrence.

This study is not without limitations. While we showed that the multitask AI-based model outperformed current methods used in surgical practices for risk assessment and provided predictions with good all-around performance in evaluating malignant progression and lesion recurrence among OPMD patients, future studies should aim to improve the external testing of the model presented in this study with data from more centers and regions. Additionally, the model’s application may be improved by identifying an optimal probability threshold/cutoff for binary stratification of cancerization and lesion recurrence risks, which should be determined from prospective studies to calculate additional metrics such as the sensitivity and precision of the model. We will continue to expand on the training samples to assess the effect of model updating on overall performance.

## Conclusions

Overall, this study successfully developed a multitask AI-based model using TabPFN and multidimensional data to adequately predict the risk of treatment failure, malignant progression, and lesion recurrence among patients with OPMD treated via surgical excision. The model outperformed the current methods of risk assessment (WHO and binary dysplasia grading) in discriminative ability and calibration, with superior potential clinical benefits. With further model updating and external testing, the model holds the potential for comprehensive assessment and data-driven decision support for surgeons involved in the management of OPMD.

## Supplementary Material

## Data Availability

The datasets generated during and/or analysed during the current study are not publicly available due to the need to maintain patient confidentiality but are available from the corresponding authors on reasonable request.
