# Supplementary material for: Artificial intelligence for predicting post-excision recurrence and malignant progression in oral potentially malignant disorders: a retrospective cohort study
Source: Int J Surg. 2025 Oct 7;112(1):1392–401. doi: 10.1097/JS9.0000000000003592 (PMC12825756; doi:10.1097/JS9.0000000000003592)
Supplement: Supplementary file 1 [file js9-112-1392-001.docx]

**Supplementary Table 1:** Hyperparameters selected for model training

| Random Forest | XGBoost | TabNet |
| --- | --- | --- |
| Estimators: 100 | Estimators: 650 | Batch size: 16 |
| Maximum depth: 1 | Maximum depth: 1 | Learning rate: 0.001 |
| Class weights = *balanced* | Learning rate: 0.01 | Epochs: 100 |
|  | Subsample: 0.7 | Early stopping patience: 10 |
|  | Scale positive weights = 2.18367 | Optimizer: Adam |
|  |  | Class weights = *balanced* |

**Supplementary Figure 1:** Receiver operating characteristic curve indicating the comparative performance of XGBoost, RF, and TabNet models in predicting surgical treatment failure (A,B,C), malignant progression (D,E,F), and lesion recurrence (G,H,I).


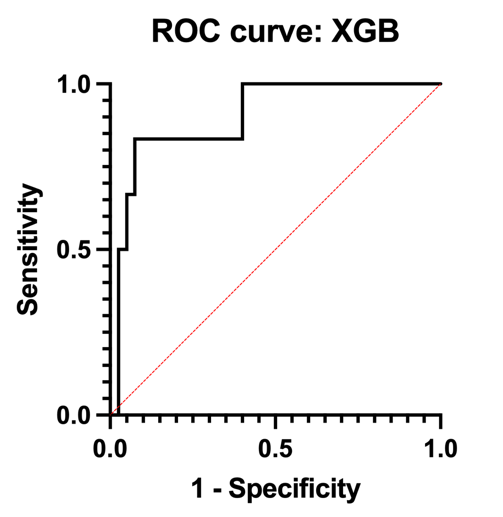

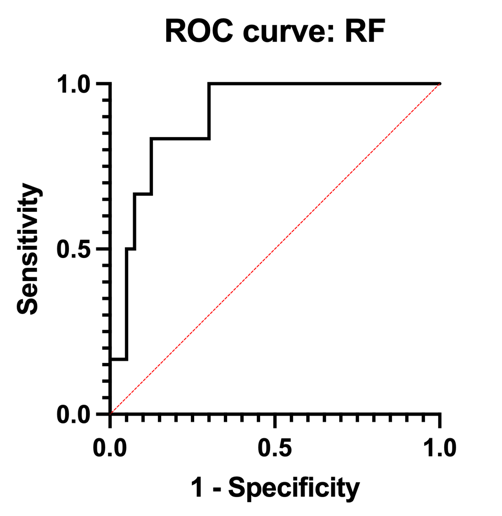

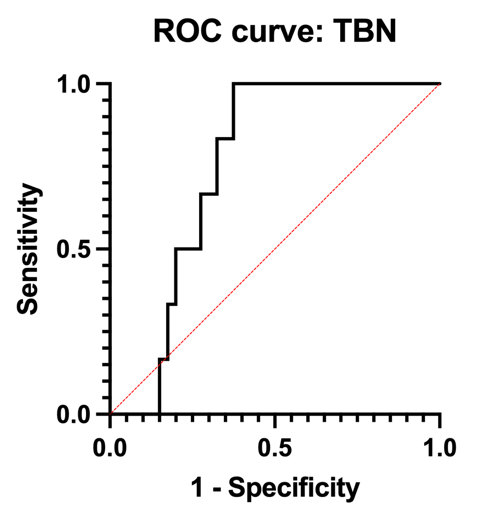

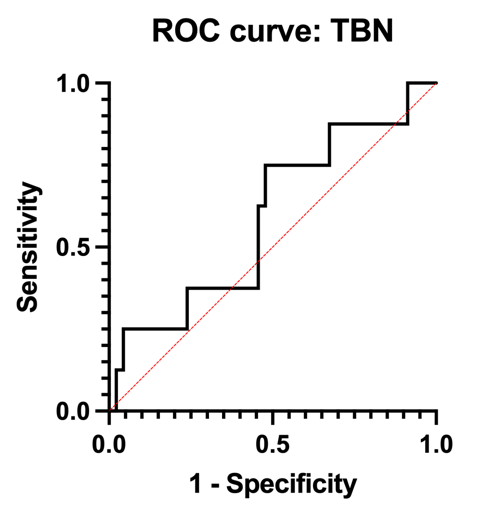

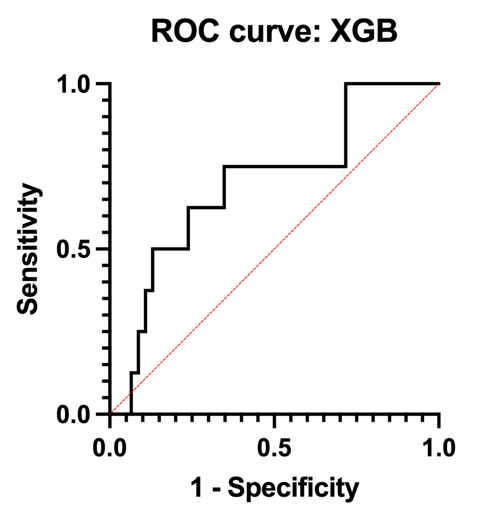

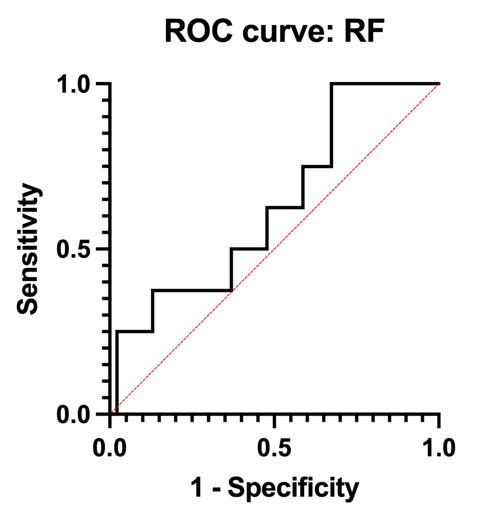

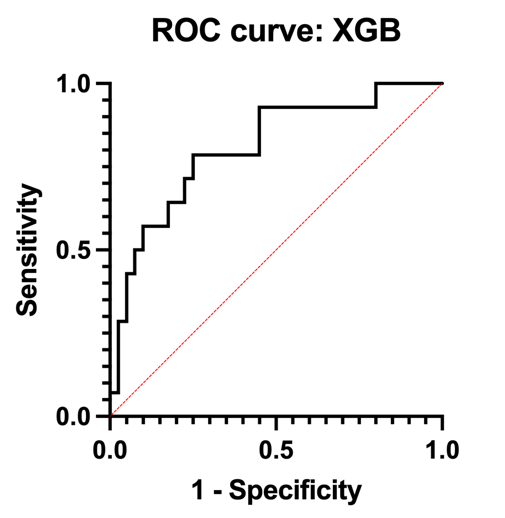

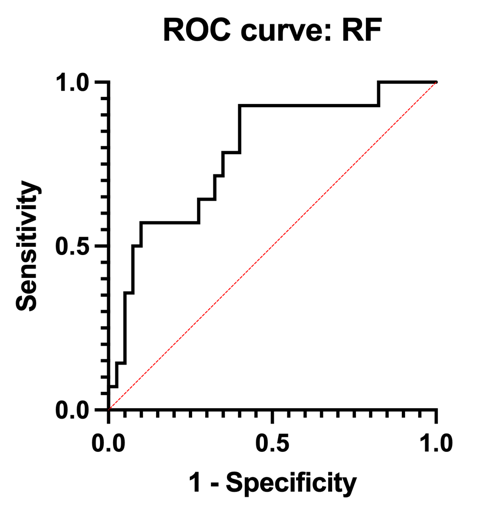

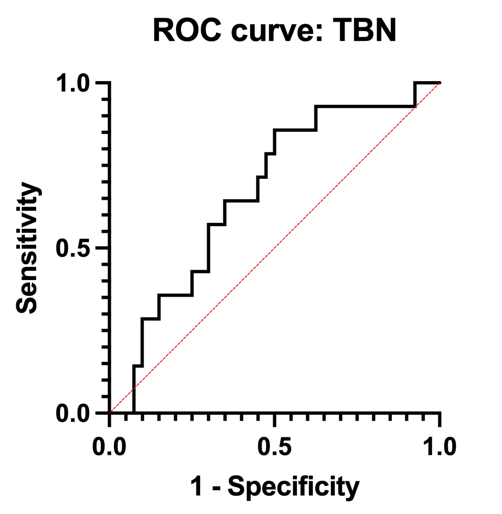


**A**

**B**

**C**

**D**

**E**

**F**

**G**

**H**

**I**
